# Electrochemical and biosensor techniques to monitor neurotransmitter changes with depression

**DOI:** 10.1007/s00216-024-05136-9

**Published:** 2024-01-30

**Authors:** Kelly E. Dunham, B. Jill Venton

**Affiliations:** Department of Chemistry, University of Virginia, Charlottesville, VA 22904 USA

**Keywords:** Cytokine, SSRI, Ketamine, Chronoamperometry, Fast-scan cyclic voltammetry, Biosensors

## Abstract

Depression is a common mental illness. However, its current treatments, like selective serotonin reuptake inhibitors (SSRIs) and micro-dosing ketamine, are extremely variable between patients and not well understood. Three neurotransmitters: serotonin, histamine, and glutamate, have been proposed to be key mediators of depression. This review focuses on analytical methods to quantify these neurotransmitters to better understand neurological mechanisms of depression and how they are altered during treatment. To quantitatively measure serotonin and histamine, electrochemical techniques such as chronoamperometry and fast-scan cyclic voltammetry (FSCV) have been improved to study how specific molecular targets, like transporters and receptors, change with antidepressants and inflammation. Specifically, these studies show that different SSRIs have unique effects on serotonin reuptake and release. Histamine is normally elevated during stress, and a new inflammation hypothesis of depression links histamine and cytokine release. Electrochemical measurements revealed that stress increases histamine, decreases serotonin, and leads to changes in cytokines, like interleukin-6. Biosensors can also measure non-electroactive neurotransmitters, including glutamate and cytokines. In particular, new genetic sensors have shown how glutamate changes with chronic stress, as well as with ketamine treatment. These techniques have been used to characterize how ketamine changes glutamate and serotonin, and to understand how it is different from SSRIs. This review briefly outlines how these electrochemical techniques work, but primarily highlights how they have been used to understand the mechanisms of depression. Future studies should explore multiplexing techniques and personalized medicine using biomarkers in order to investigate multi-analyte changes to antidepressants.

**Graphical abstract:**

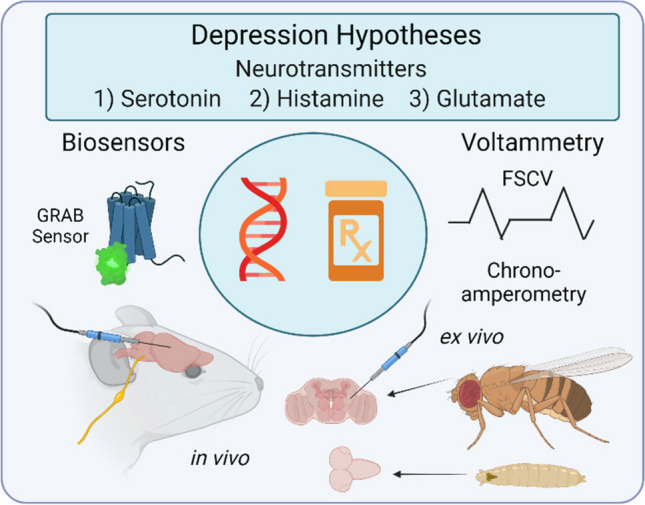

## Introduction

**Fig. 1:**
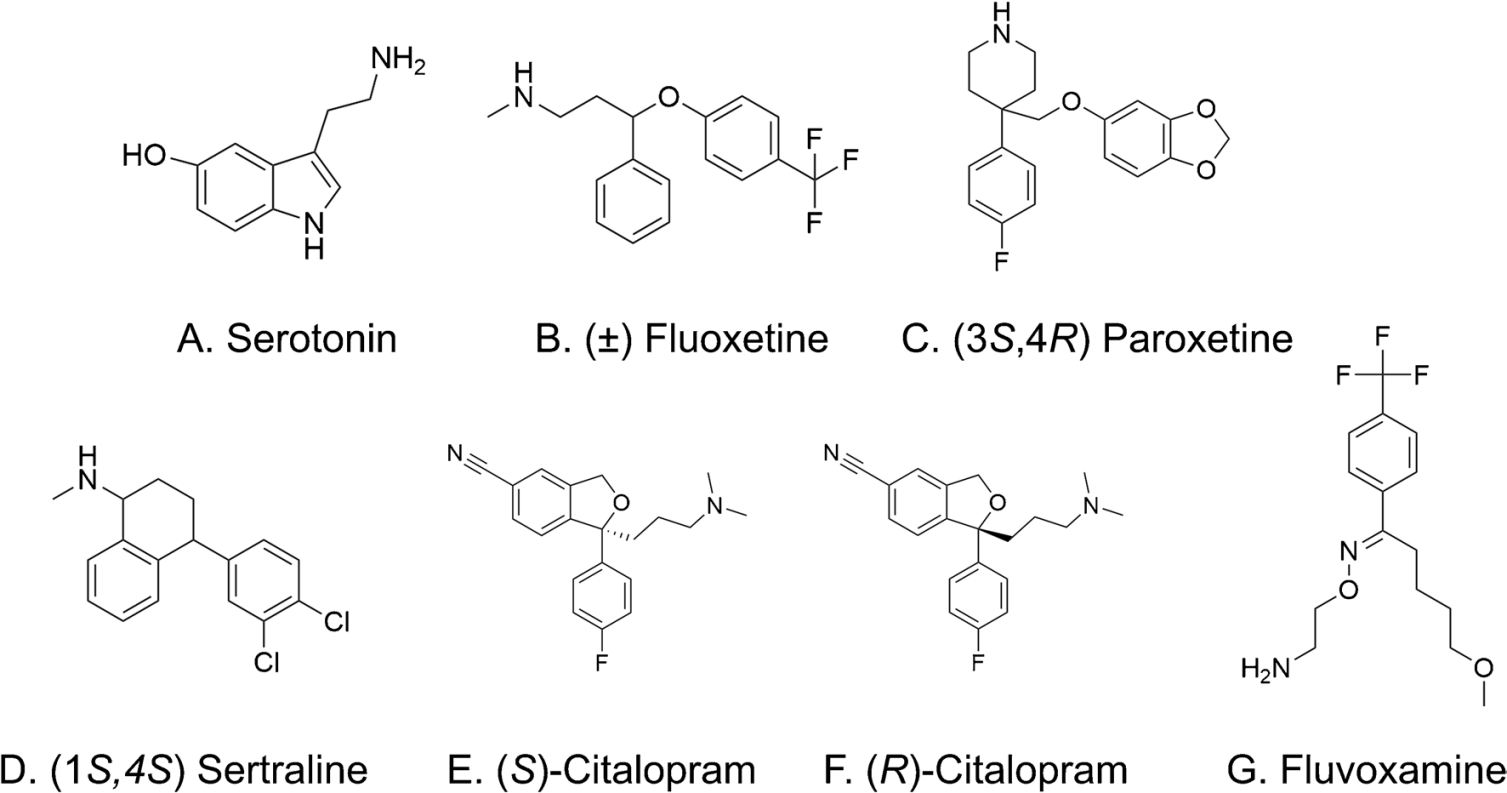
Commonly prescribed SSRI antidepressants and their chemical structures. **A** Serotonin is an indolamine monoamine neurotransmitter whose precursor is the amino acid tryptophan. **B** Fluoxetine (Prozac) was the first SSRI and shows a similar chemical structure to diphenhydramine (Benadryl). **C** Paroxetine (Paxil), **D** sertraline (Zoloft). **E** (*S*)-Citalopram is the S-enantiomer of citalopram and is exclusively produced as escitalopram (Lexapro). **F** (*R*)-Citalopram is the (R)-enantiomer of citalopram. Citalopram (Celexa) is a racemic mixture of the (*S*) and (*R*)-enantiomers. **G** Fluvoxamine (Luvox)

Depression is a common mental illness that affects hundreds of millions of people in the world, and 1 out of 5 adults in the USA has been treated for depression [1]. Depression causes overwhelming feelings of sadness and loss of interest that manifest in a range of symptoms, with adverse changes to sleep, appetite, and energy, as well as thoughts of suicide [2]. Likewise, depression is commonly diagnosed with other illnesses, such as anxiety and eating disorders [3]. Before the 1980s, the most common antidepressants included tricyclic amines (TCAs) and monoamine oxidase inhibitors (MAOIs), which were dangerous and known to cause heart issues [4]. In the early 1970s, scientists found in autopsies of suicide patients that brain structures rich in serotonin, such as the dorsal raphe nuclei and hippocampus, were smaller or had atrophied [4]. This discovery led to the monoamine hypothesis of depression that suggests low concentrations of serotonin create depression symptoms [4]. In 1987, fluoxetine hydrochloride (Prozac) was the first approved selective serotonin reuptake inhibitor (SSRI) antidepressant that blocked serotonin clearance through serotonin transporter (SERT) inhibition [4]. However, its efficacy is extremely variable from person to person. Over the following decades, a second generation of SSRIs was introduced, including paroxetine (Paxil), sertraline (Zoloft), citalopram (Celexa), escitalopram (Lexapro), and fluvoxamine (Luvox) [5]. Although all SSRIs bind to SERT, they possess very different chemical structures, which are illustrated in Fig. 1A–G [5], and elicit different downstream changes in serotonin reuptake and release that are not well understood. Additionally, genetic differences in SERT may also contribute to their inconsistent efficacies [6, 7]. Unfortunately, around 50% of people who take antidepressants do not improve, and some try two or more SSRIs [8]. This type of depression is classified as treatment-resistant depression (TRD) [9], and people diagnosed with TRD have a higher risk of suicide [8]. However, direct measurements of neurotransmitters using electrochemical techniques and biosensors are useful to understand the neurotransmitter control of depression and could lead to new treatments.

New hypotheses for neurotransmitter dysregulation during depression are being developed because of variable efficacies with drugs that target serotonin. An inflammation hypothesis for depression suggests that rising histamine levels decrease serotonin and increase cytokines [10–12]. Low levels of tryptophan, which is the precursor to serotonin, act as a signal that upregulates pro-inflammatory cytokines and increases oxidative stress that disrupts DNA, protein, lipid membrane, and cell synthesis [10]. Interestingly, fluoxetine was initially designed from diphenhydramine (Benadryl), a classic antihistamine, and may also target histamine [4]. Still, it is not clear how serotonin and histamine change in real-time or mediate inflammation and depression, and analytical techniques that monitor neurotransmitter changes with depression would better elucidate these mechanisms that regulate it [13–15].

Glutamate is another neurotransmitter that is hypothesized to be disrupted in depression and could be a target for antidepressants. Trullas and Skolnick first suggested glutamate modulation with NMDA antagonists, like ketamine, could be used as antidepressants, since a lack of glutamate potentiation in the hippocampus is linked to stress [16]. Early clinical trials also showed sub-anesthetic doses of ketamine alleviated depression symptoms [17, 18], and were replicated in larger, placebo-controlled trials in the 2010s [18]. However, the full neurotransmitter mechanisms of ketamine are unknown, and whether they only work through glutamate or also interact with other systems, including serotonin, is unclear [19, 20].

Over the past several decades, analytical chemists developed techniques to answer questions concerning how neurotransmitters change with diseases, such as depression, and with treatments like SSRIs and ketamine. Microdialysis was first used to sample brain fluids that were analyzed with liquid chromatography. However, the probes are large and the time scale of measurements is slow, typically on the order of minutes [21]. In the 1960s, Ralph Adams was the first to use electrochemical sensors to monitor small electroactive neurotransmitters in rat brain tissue [22, 23]. Real-time electrochemical techniques, including fast-scan cyclic voltammetry and chronoamperometry, were developed to directly measure electroactive neurotransmitters quickly, on a sub-second time scale [24–26]. In addition to direct electrochemistry, biosensors have also been created that feature enzymes specific for detection of non-electroactive neurotransmitters, like glutamate [27]. Newer fluorescent sensors that use G-protein-coupled receptors (GPCRs) and bacterial proteins also act as fluorescent reporters to directly image neurotransmitters on the cell surface [28, 29]. Additionally, these techniques aid in understanding real-time neurotransmitter changes in small volumes or in discrete areas of the brain, since their electrode sizes are much smaller than microdialysis probes and the time scale is much faster [21]. Ultimately, this review describes these analytical techniques for neurotransmitter detection and outlines how they have been used to investigate the neurotransmitter hypotheses of depression. Together, they will continue to be used in the future to enable depression research to investigate mechanisms of treatments.

## Electrochemical techniques to measure neurotransmitters

### Chronoamperometry

Chronoamperometry is a voltammetry technique that steps to a potential sufficient to oxidize or reduce a neurotransmitter. The current measured is proportional to the concentration of analyte according to Faraday’s law [21, 22]. For serotonin, the Daws group steps from a potential of 0 V to +0.55 V every 100 ms to obtain oxidative and reductive currents, and a ratio of oxidative to reductive current [30, 31]. With microelectrodes, it is also possible to measure neurotransmitter release from a single cell [24, 32]. Even though chronoamperometry shows real-time neurotransmitter detection, it cannot distinguish between neurotransmitters when high oxidation potentials are applied (i.e., serotonin and histamine could both be measured at +0.6 V) [22]. Thus, for selectivity, neurotransmitters may be measured in specific brain regions without other interferents [21–23], or specific electrode surface-coating polymers may be used to shield unwanted analytes [33].

### Fast-scan cyclic voltammetry

**Fig. 2:**
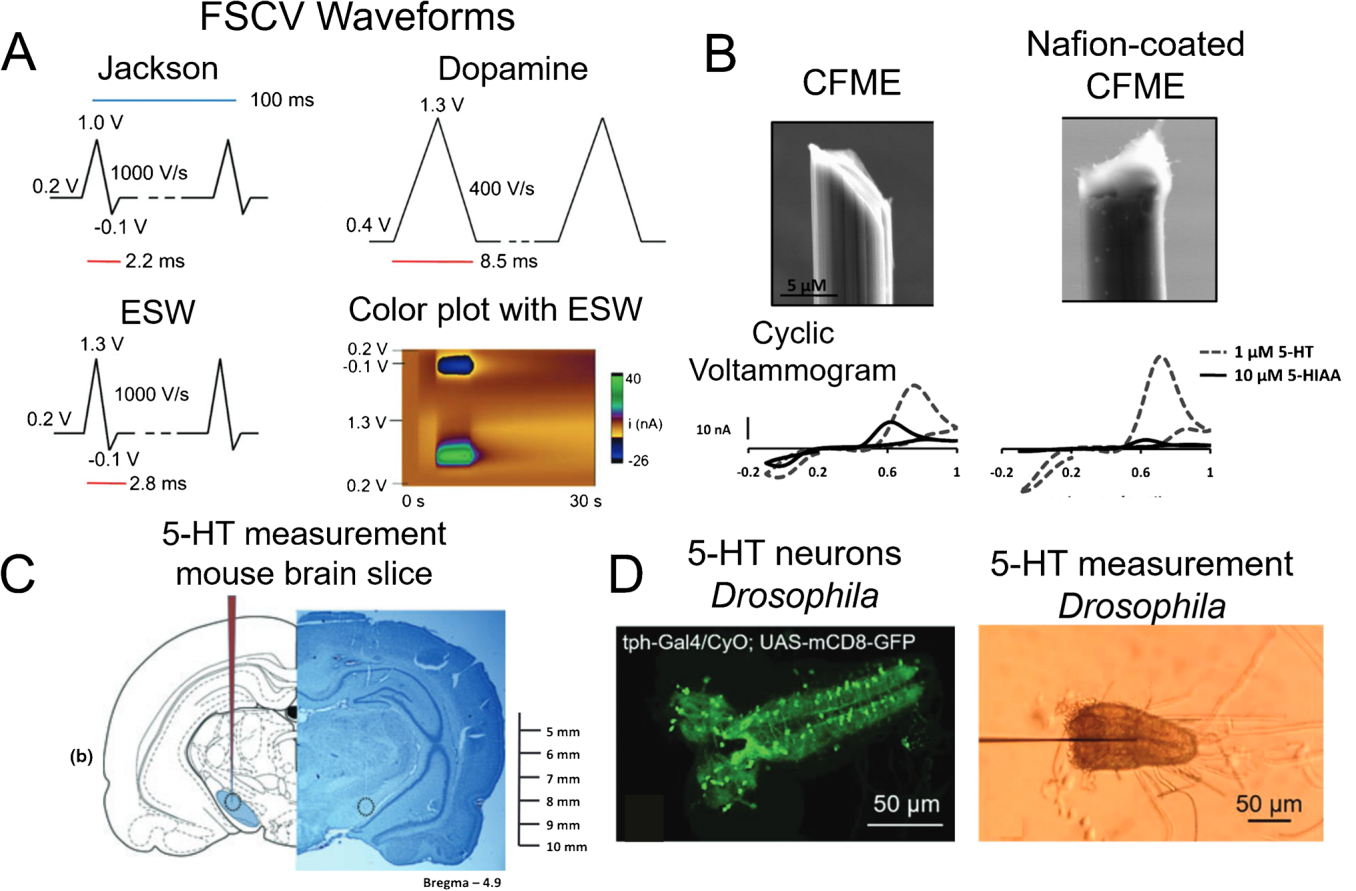
Fast-scan cyclic voltammetry (FSCV) to measure real-time serotonin changes. **A** Tool-kit of FSCV waveforms measure serotonin with ms temporal resolution. From clockwise, Jackson waveform, dopamine waveform, and extended serotonin waveform (ESW). Color plot shows changes in current detected overtime with the ESW. Serotonin oxidation is in green, while reduction is in blue. Current detected is proportional to 5-HT concentration (permission from Dunham and Venton 2020, *Analyst*). **B** Carbon fiber microelectrodes (CFMEs) bare and with Nafion-coating. Nafion increases serotonin sensitivity and decreases electrode fouling to 5-HIAA in mice brain slices (permission from Hashemi et al. 2009, *Analytical Chemistry*). **C** CFME placement in mouse brain slice for 5-HT measurement in substantia nigra and mPFC (permission from Hashemi et al. 2011, *Journal of Neurochemistry*). **D** *Drosophila melanogaster* (fruit fly) shows similar 5-HT machinery to mammals. GFP imaging shows 5-HT neurons in *Drosophila* larvae ventral nerve cord tissue, and microscope image shows optimal CFME placement to measure with FSCV

Fast-scan cyclic voltammetry (FSCV) is another popular electrochemical technique that applies a linear ramp potential, known as a voltage waveform, with high scan rates of 100–1000 V/s (Fig. 2A) [22, 23]. FSCV is commonly used with a carbon fiber microelectrode (CFME) to measure neurotransmitters in brain tissue with millisecond temporal resolution. Compared to chronoamperometry, FSCV possesses higher selectivity for neurotransmitters like serotonin, because it produces a distinctive cyclic voltammogram (CV). In the 1990s, Wightman’s group was the first to design specific waveforms for both serotonin [34] and histamine detection [35]. However, oxidation of both serotonin and histamine creates oxidative byproducts that can polymerize to the electrode and cause electrode fouling [34, 36]. To ameliorate this issue, surface coatings and waveform modifications that lessen fouling have improved their detection over the past decade [36, 37].

Additionally, the Wightman group discovered that Nafion-coating a CFME disk electrode eliminated electrode fouling to serotonin and its downstream metabolite [36], 5-HIAA (Fig. 2B), in mice brain slices (Fig. 2C). Nafion, which is negatively charged, also increases sensitivity to positively charged serotonin, and Nafion-coated electrodes have higher detected currents [36]. The Venton lab also created a tool-kit of serotonin waveforms that improved detection with bare CFMEs [37]. The triangular “dopamine” waveform did not foul with serotonin or 5-HIAA because of its negative holding potential, while the classic “Jackson” waveform fouled electrodes severely. Better methods for detection of the metabolite 5-HIAA are useful because it could be a possible biomarker for depression that could also be monitored. A new extended serotonin waveform was also developed with a higher switching potential than the Jackson waveform (+1.3 V), which increased serotonin sensitivity with decreased electrode fouling. The electrode did not foul with repeated serotonin measurements in *Drosophila melanogaster*, the fruit fly, which may be a good model for rapid serotonin screenings with pharmacology because they possess similar serotonin machinery (Fig. 2D), but do not possess monoamine oxidase-A that catalyzes serotonin’s breakdown into 5-HIAA [37, 38].

**Fig. 3:**
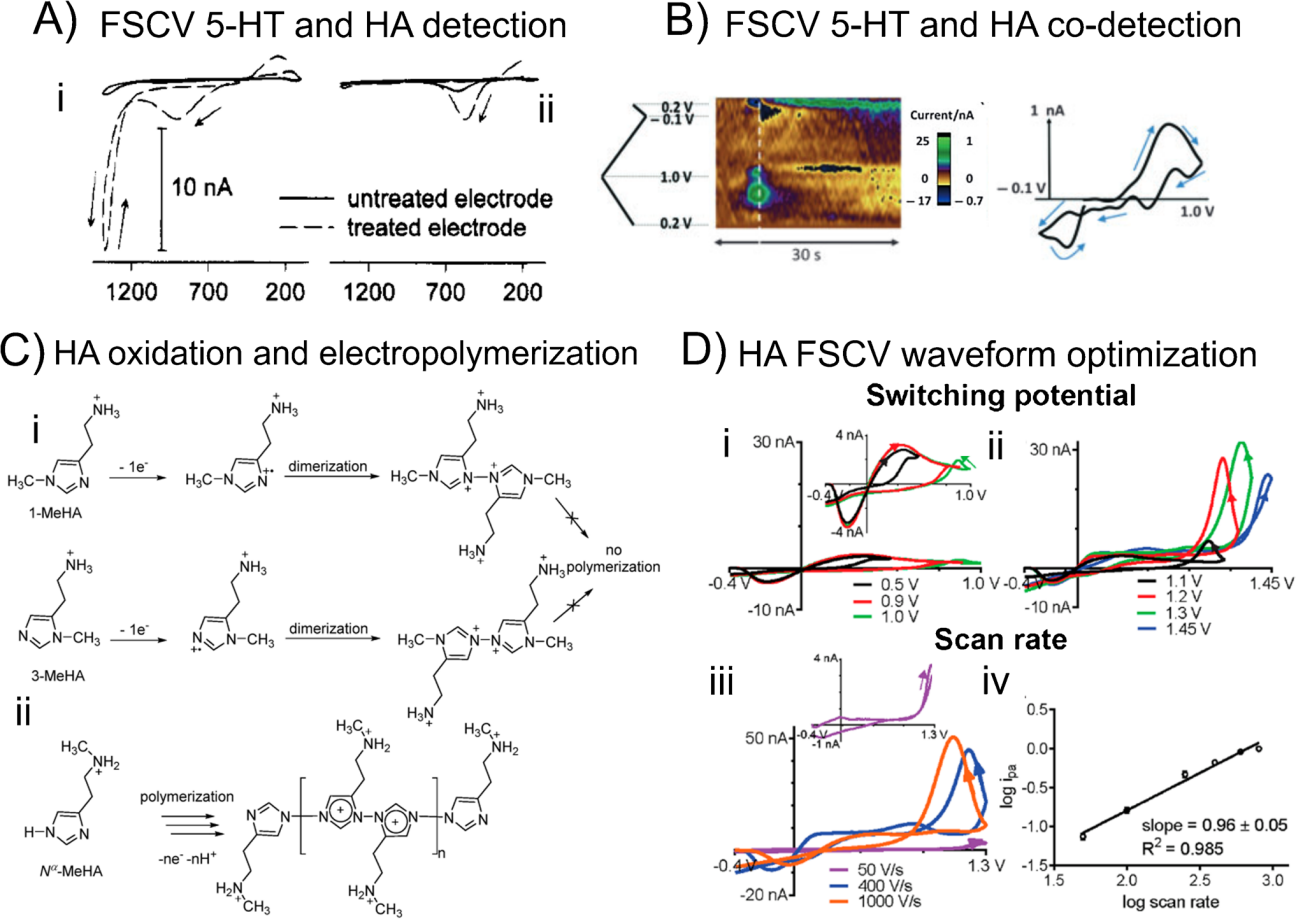
Optimization of FSCV waveforms to measure histamine (HA). **A** Detection of histamine and serotonin (5-HT) with an expanded +1.3 V switching potential (permission from Pihel et al. 1995, *ACS Analytical Chemistry*). **B** The Jackson waveform used for 5-HT detection can also co-detect histamine in vivo in mice (permission from Hashemi et al. 2011, *Journal of Neurochemistry*). **C** (i) Oxidation of 1-MeHA and 3-MeHA to determine its electropolymerization mechanism (shown in ii). Permission from Puthongkham et al. 2019, *ACS Analytical Chemistry. ***D** HA FSCV waveform switching potential and scan rate optimization for primary and secondary oxidation characterization

In addition to serotonin FSCV detection, several groups have designed different waveforms for histamine detection [39–42]. Initially, the Wightman lab showed that histamine’s oxidation peak was different from serotonin [42], and could be measured with a triangular waveform from +0.1 V to +1.3 V back to +0.1 V at 800 V/s using CFMEs. Serotonin oxidized around +0.6 V, while histamine’s oxidation peak was closer to +1.3 V (Fig. 3A). They measured both histamine and serotonin release from a single mast cell [42]. However, in 2011, they expanded upon this work and found that histamine could be co-detected with serotonin in a single CV using the Jackson waveform and Nafion-coated CFMEs (Fig. 3B) [40]. Still, this work was controversial since the potential sweep to only +1.0 V is not sufficient to oxidize histamine. In addition to this work, the Hashemi lab also used other waveforms, including a novel (−0.5 V to −0.7 V to +1.1 V to −0.5 V) potential sweep [41], which they optimized in order to mathematically model kinetics for both analytes. The Venton group further determined the mechanism of histamine oxidation [39], which includes radical formation and dimerization, as well as electropolymerization onto CFMEs to severely foul them (Fig. 3C). In addition, a switching potential ≥ +1.1 V is required to oxidize histamine (Fig. 3D), and Nafion-coating CFMEs helps alleviate CFME fouling. Overall, there are many different waveforms and proposed mechanisms for histamine oxidation, but CFMEs can be used to determine histamine concentration in vivo.

### Biosensors

**Fig. 4:**
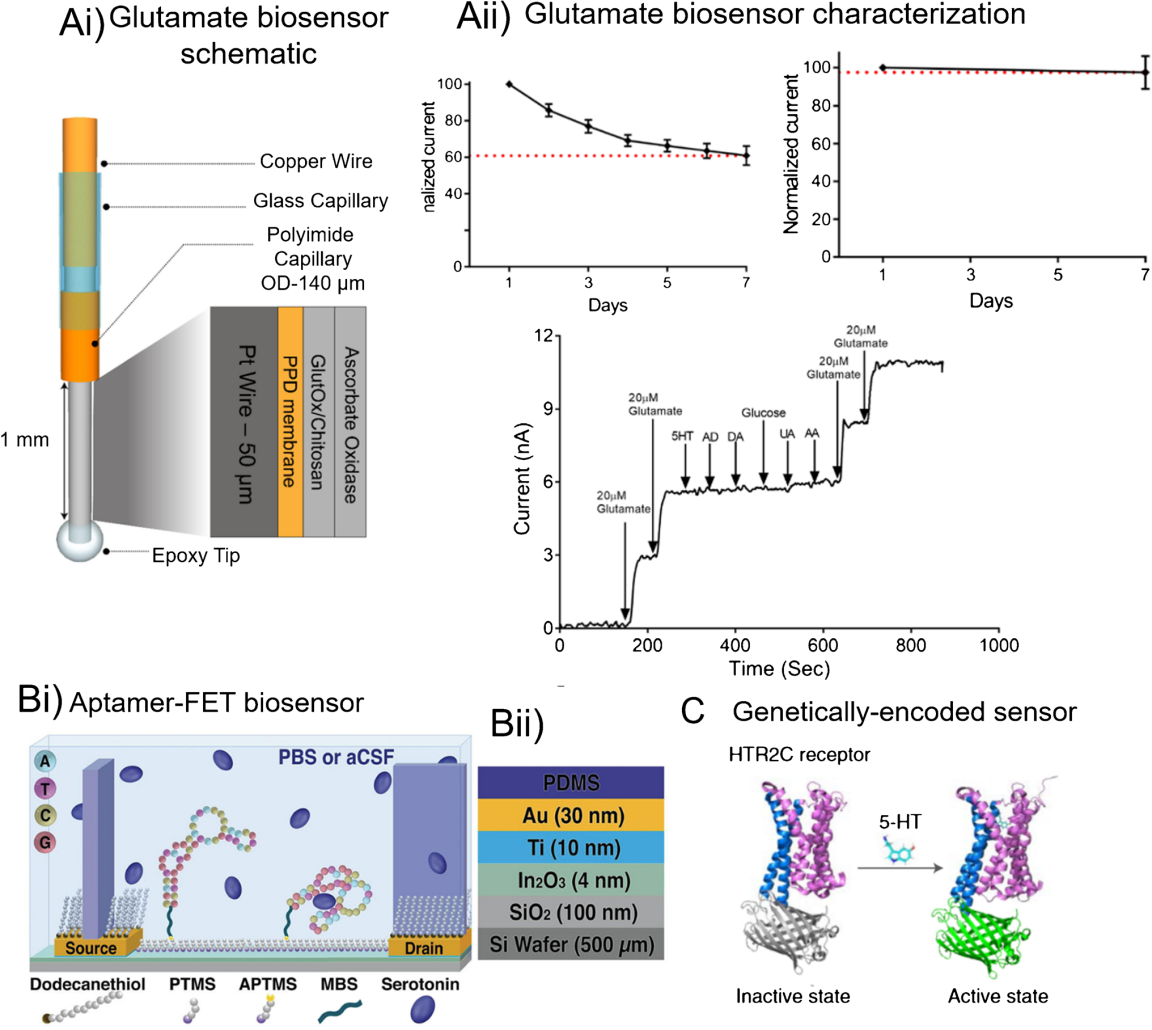
Biosensors used for depression research. **A** (i) Schematic of a glutamate biosensor for in vivo measurements that shows high selectivity and stability (ii). Permission from Ganesana et al. 2019, *Biosensors and Bioelectronics*. **B** (i) Aptamer-FET biosensor schematic with (ii) biosensor layer deposition (permission from Nakatsuka et al. 2018, *Science*). **C** Genetically encoded sensor for serotonin based on a 5-HT_2C_ receptor with its GFP in the inactive and active state for real-time imaging (permission from Wan et al. 2021, *Nature Neuroscience*)

In order to detect real-time changes in small biomolecules, biosensors commonly use a biological macromolecule attached to a sensor to produce electroactive intermediates that are easily quantifiable with electrochemistry [43]. Formally, there are several categories of biosensors that are distinguished by the electrochemical technique (i.e., amperometry, voltammetry, electrochemical impedance, etc.) or by biological component (i.e., proteins, peptides, antibodies, or DNA) [43]. Biosensors are extremely sensitive and selective for their analyte of interest. An example is a glutamate oxidase biosensor, which is attached to an electrode surface and binds glutamate (non-electroactive) and catalyzes its breakdown into ɒ-ketoglutarate, ammonia, and hydrogen peroxide that is measured with electrochemistry [44]. Recently, the Venton lab created a micro-biosensor for glutamate with a polymer coating on a Pt wire only 50 µm in diameter (Fig. 4A) [44]. They measured glutamate with amperometry in mouse brain slices and that their sensor was stable for over a week with repeated measurements.

For depression research, aptamer biosensors are commonly used to measure serotonin changes (Fig. 4B) [45, 46]. Aptamer biosensors contain short lengths of DNA or RNA that selectively bind small molecule targets, such as neurotransmitters (serotonin) or medications (antidepressants, antibiotics) [45, 46]. Typically, they are designed on gold (Au) surfaces where the DNA probe easily binds to thiol groups and contains an electroactive reporter molecule that produces a signal once the target molecule binds and creates a conformation change [45]. More recently, aptamers have also been integrated into field-effect transistor (FETs) sensors that typically contain a source that flows solution containing the target molecule to a drain and has aptamers strategically placed between them to attach and measure the analyte [45]. This also creates a charge current, which can be subtracted to measure low concentrations of analyte and can be easily integrated into a MEMs device. FETs have high sensitivity (picomolar ranges), much higher than voltammetry techniques such as FSCV. However, the biological component of the sensor limits the sensor’s shelf-life [44, 45].

An alternative to electrochemical biosensors is genetically encoded fluorescent sensors (GES) [29]. These sensors genetically tag a GFP fluorescent reporter to a G-protein-coupled receptor (GPCR), which will fluoresce when a neurotransmitter (serotonin, histamine) binds to it (Fig. 4C). For glutamate, periplasmic binding proteins (PBPs) from bacteria have also been used to make sensors, such as iGluSnFRs [27, 47]. G-protein-coupled receptors and PBP-type sensors are highly selective and sensitive to one neurotransmitter, and analytes do not need to be electroactive [27]. However, fluorescence data collection is limited to only 90 second periods because of photobleaching issues [48]. Similar to voltammetry, GES only show changes in fluorescence over time and cannot determine basal levels [29]. In addition, the signal from GES is not easily calibrated to concentration, which is different than chronoamperometry, FSCV, and aptamer sensors which can be calibrated to estimate concentrations.

## Three neurotransmitter hypotheses of depression: recent discoveries on the mechanisms of antidepressants

### Serotonin and SSRI antidepressants

**Fig. 5:**
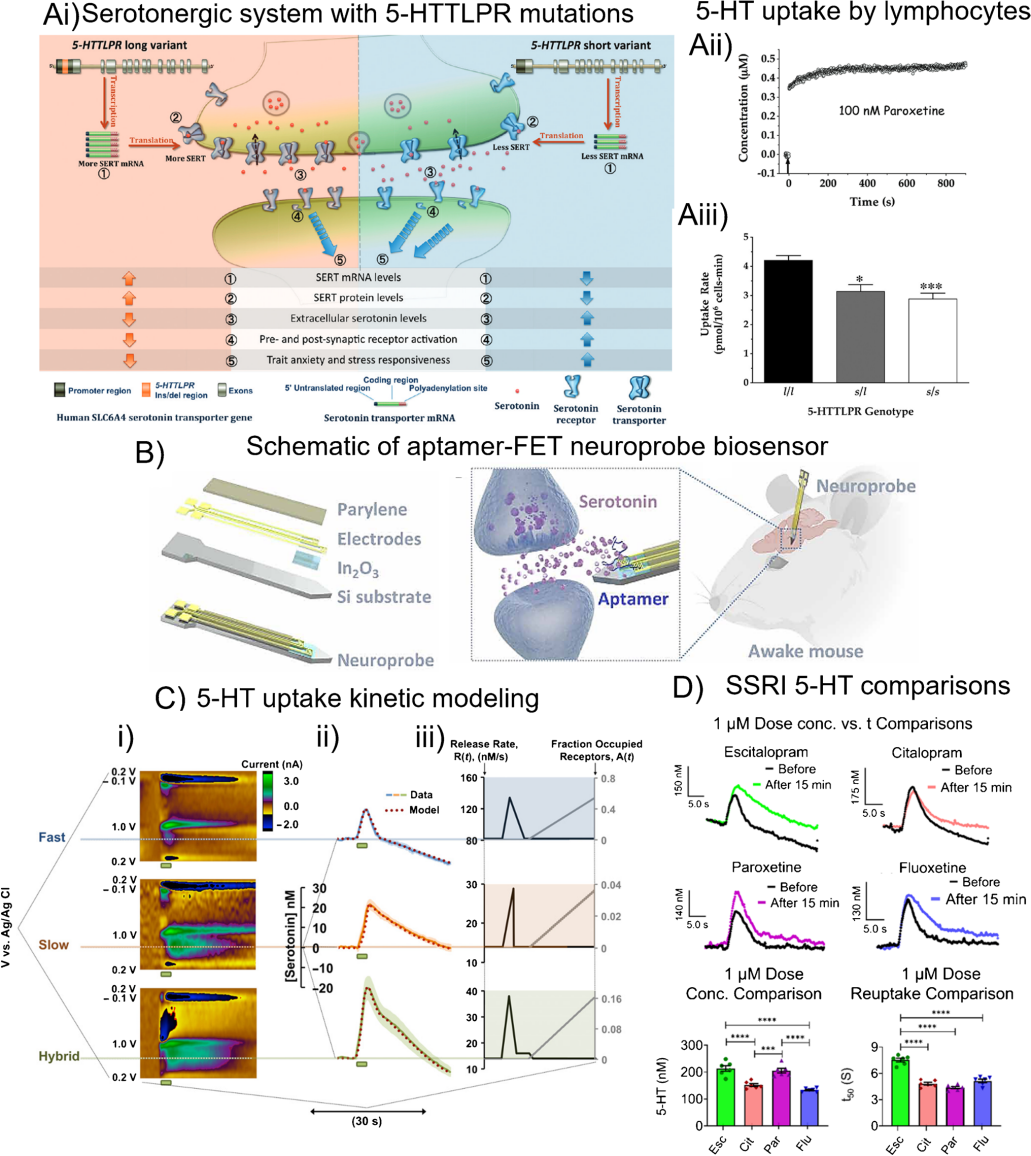
Electrochemical techniques show serotonin uptake changes with different SSRI antidepressants. **A** (i) Schematic of serotonergic system with serotonin transporters (SERTs) and serotonin receptors. Long (*l*) and short (*s*) allele variant for 5-HTTLPR and its theoretical changes to serotonin are also listed. (ii) Chronoamperometry was used to show lymphocytes (immune cells) from rhesus macaques can uptake serotonin and paroxetine inhibits uptake. (iii) (s)-Allele 5-HTTLPR shows faster reuptake of serotonin in lymphocytes compared the (*l*) variant (permission from Singh et al. 2010, *ACS Chemical Neuroscience*). **B** Schematic of the design and application of an aptamer-FET biosensor for in vivo serotonin measurement (permission from Zhao et al. 2021, *Science advances*). **C** (i–iii) FSCV representative fast, slow, and hybrid serotonin kinetic responses from stimulated serotonin release in the substantia nigra (permission from Wood et al. 2014, *Journal of Neurochemistry*). Data were used to create the free kinetic modeling program, The Analysis Kid, using FSCV. **D** FSCV data shows serotonin concentration and reuptake changes with a low, 1 µM dose of escitalopram, citalopram, paroxetine, and fluoxetine in fruit flies. Each SSRI changed serotonin differently and concentration and reuptake are coupled for some, but independent for others (permission from Dunham and Venton 2022, *Journal of Neurochemistry*)

Serotonin (5-hydroxytryptophan, 5-HT) is a monoamine neurotransmitter that regulates many physiological functions [1], and is phylogenetically conserved in several species, including humans, mice, and fruit flies. Serotonin is synthesized from the diet-derived amino acid tryptophan, which travels throughout the blood to act as a clotting factor with wound healing. Remarkably, most serotonin is located in the gut in enterochromaffin cells, although correlations of gut serotonin and depression are not well established. Brain serotonin is created from tryptophan, which crosses the blood-brain barrier and enters the dorsal raphe nuclei (DRN) of the brain stem [49]. Primarily, serotonin cell bodies radiate from the DRN to the substantia nigra, hypothalamus, nucleus accumbens, and medial prefrontal cortex [49, 50], and these diverse signaling pathways allow serotonin to regulate different behaviors like sleep, mood, memory, and appetite [50, 51]. The serotonin transporter (SERT) provides a negative feedback loop to reuptake extracellular serotonin back into the neuron to clear released serotonin, which is shown in Fig. 5A [1, 50]. After reuptake, excess serotonin is either re-packaged into vesicles for release again or destroyed by lysosomes [52]. Additionally, there are seven classes of serotonin receptors in mammals that regulate serotonin neuronal activation and inhibition [1, 50]. All are ligand-gated ion channel GPCRs, except 5-HT-3 receptors. The GPCR serotonin receptors are also illustrated in Fig. 5A [1, 50]. These receptors act as either autoreceptors or heteroreceptors. Autoreceptors are located on presynaptic neurons and attenuate serotonin release with a negative feedback loop, while heteroreceptors are on post-synaptic neurons and regulate serotonin through postsynaptic feedback [1, 50]. In addition to mammals, *Drosophila melanogaster* also possess SERT (dSERT) and serotonin receptors that show homology with structure and function [53–55]. Although a controversial new article suggests that serotonin is not involved in depression, decades or work have shown changes with serotonin with both genetic manipulations and antidepressants [56]. Specifically, these bioanalytical techniques that measure serotonin help identify how SERT and serotonin receptors control serotonin signaling in the brain, which gives insight into how their dysfunction causes depression, anxiety, and aggression.

With chronoamperometry, the Andrews lab explored several fundamental changes to the serotonin system with genetic mutations and antidepressants [57, 58]. For example, they explored the effect of a 50% SERT knockdown (SERT +/-) in mice versus a complete SERT knockout (SERT-/-) on serotonin clearance rates in brain liposomes or synaptosomes [57]. Serotonin clearance rates increased with increased oxygen. Also, decreased SERT expression increased uptake of serotonin, which was confirmed with paroxetine (1 µM) treatments in control SERT +/+ mice [57]. Likewise, serotonin uptake rate changed with a short-allele SERT variant in rhesus macaques. In mammals, a short (*s*) 5-HTTLPR allele variant decreased mRNA and protein expression of SERT, while the long (*l*) allele did the opposite, and changed basal serotonin concentrations (Fig. 5A) [58]. They used a novel boron-doped diamond microelectrode, which decreased electrode fouling to serotonin with chronoamperometry. Additionally, lymphocytes actively cleared serotonin and serotonin uptake was inhibited by SSRIs, like paroxetine (Fig. 5Aii). The (*s*) 5-HTTLPR allele showed faster serotonin clearance compared to the (*l*) allele variant, and the homozygous (*s*) allele had faster reuptake of serotonin than a heterozygous allele (Fig. 5A). Together, these data suggest that the (*s*) 5-HTTLPR allele variant reuptake more quickly, which may alter serotonin release mechanisms differently to maintain higher basal serotonin concentrations. Recently, the Andrews lab has also created an implantable aptamer-FET neuroprobe biosensor that measures serotonin changes in mice in vivo, which is illustrated in Fig. 5B [59]. Since this technique is very new, it has not been characterized extensively, but it will allow for the real-time measurement of serotonin to understand depression behavior and treatments in the future. Thus, analytical techniques for measuring serotonin have shown different serotonin levels with different serotonin genotypes, which gives insight into why some patients may respond differently to different antidepressants.

Also with chronoamperometry, the Daws group studied serotonin clearance and antidepressant mechanisms [31, 33, 60]. Like many fundamental studies of serotonin uptake, these studies were performed in anesthetized animals, which are easier to study than freely moving animals and have similar clearance mechanisms, even under anesthesia. They first characterized fundamental uptake changes with SERT and the norepinephrine transporter (NET) in mice using fluvoxamine and citalopram, and found that both SERT and NET contribute to the active clearance of serotonin in the CA3 region of the hippocampus, which is a very important region studied in depression [9, 60]. They later expanded on this work to explore reuptake serotonin kinetics that were first described by Shashkan and Snyder in the 1970s, specifically “uptake-1” and “uptake-2” of serotonin [61, 62]. Uptake-1 is high affinity for serotonin with slow transport kinetics, while uptake-2 is low affinity with faster transport kinetics. Ultimately, the Daws group used pharmacology to show that low affinity transporters, like organic cation transporters (OCT) and plasma membrane monoamine transporters (PMAT) play important roles in serotonin reuptake [33, 61, 63]. This is an important finding, as it shows that clearance is not just SERT dependent, and that other transporters may be targets for antidepressants as well.

The Daws lab also investigated genetic and modulatory changes to SERT with serotonin receptors and antidepressants [30, 31, 64, 65]. Initially, they explored serotonin changes with male 5-HTT (SERT) KO mice (+/+, +/-, and -/-) [31], and found that serotonin clearance decreased in the heterozygous (+/-) group, and even more in the homozygous (-/-) mice. The SSRI fluvoxamine both inhibits reuptake of serotonin and increases concentrations through SERT, since the (-/-) mice did not change serotonin clearance or concentrations. Likewise, with escitalopram and SERT KO mice, radio-ligand binding assays of [H^3^]-citalopram show SERT is required for antidepressants to function [65]. They also examined SERT knockout (-/-) and 5-HT_1B_ autoreceptor genotypes [30], and chronoamperometry showed SERT activity did not change with different 5-HT_1B_ mutations in the CA3 region of the hippocampus. However, they found that the 5-HT_1B_ receptor antagonist cyanopindolol did not inhibit serotonin in the 5-HT_1B_ (-/-) or SERT (-/-) mice, which was different from the wild type and suggests that both work together to regulate serotonin inhibition. Ultimately, analytical methods here add functional information about how SERT and 5-HT_1B_ work together in various genotypes with SSRIs.

With FSCV, the Hashemi lab simultaneously measured serotonin and dopamine changes in vivo in rats to understand their dynamic changes in clearance with reuptake inhibitors, as well as vesicular monoamine transporter (VMAT) inhibitors [66]. Dopamine release was more sensitive to changes in vesicular packaging, while serotonin was tightly controlled by SERT through reuptake. Michaelis-Menten kinetics changes after SSRI antidepressants, citalopram and escitalopram, were also similar to previous work by Shaskan and Snyder and the Daws group [67, 68]. The Hashemi lab also created classical models for uptake-1 (fast) and uptake-2 (slow) with antidepressants. However, they saw serotonin traces with escitalopram show “hybrid” clearance of serotonin that starts fast and then slows at low, antidepressant doses [62]. These uptake responses are illustrated in Fig. 5C with FSCV color plots, concentration versus time plots, and release and fraction retained rates. They also used a form of FSCV, called fast-scan cyclic adsorption voltammetry, in order to measure basal levels of serotonin in mice, and saw that females typically have lower concentrations of serotonin compared to males [68]. Further, their models are now available in a web-based tool, known as the Analysis Kid [69], with their previously collected mathematical models for serotonin and dopamine. This software allows users to directly upload current vs. time trace files or color plots to determine Michaelis-Menten kinetic variables for their data. It is important to note that dopamine has two types of uptake (fast and slow), while serotonin has three (fast, slow, and hybrid). Also, even though their models were created with mammals, *Drosophila* and humans show nearly identical SERT translocation speeds, which would produce similar uptake changes [70].

In *Drosophila*, the Venton group pioneered using optogenetics, which are light-activated channels specifically expressed in certain cells, to selectively release serotonin that is measured with FSCV [71, 72]. They found the releasable pool of serotonin in *Drosophila* takes 2–5 min to replenish itself [71, 73], and both serotonin synthesis and repackaging from reuptake by *Drosophila* SERT (dSERT) were necessary to replenish the releasable pool [73]. Real-time serotonin concentration and reuptake changes were also characterized with different doses of fluoxetine, escitalopram, citalopram, and paroxetine that were bath-applied to *Drosophila* ventral nerve cord (VNC) tissue [74]. They found SSRIs differentially modulated serotonin reuptake and release based on different dSERT affinities. Specifically, paroxetine showed the highest affinity to dSERT, while fluoxetine the lowest. With escitalopram and citalopram, the *S*-enantiomer showed higher affinity to dSERT, which caused serotonin concentrations to increase and reuptake to slow at lower doses compared to citalopram. With these SSRIs, reuptake was independent of serotonin release. For instance, at low doses, fluoxetine only slowed serotonin reuptake, but did not increase concentration, while paroxetine showed high concentration increases with fast serotonin clearance. These real-time serotonin changes with low SSRI doses are shown in Fig. 5D. Ultimately, this work shows that *Drosophila* is a good model for rapid pharmacology screenings to decipher how neurotransmitters change [74, 75].

Together, these analytical techniques provide valuable insights into how serotonin changes with genetic mutations to the serotonin system and with SSRI antidepressants. Fundamentally understanding how serotonin changes with different SSRIs [74], or SERT and serotonin receptor mutations [31, 58, 65], will aid in deciphering an individual’s genetic pre-disposition to react to a specific antidepressant with predictive genomics [76]. Further, the tools created by the Hashemi group allow FSCV users to track kinetic changes with any drug that impacts serotonin or dopamine [69], which opens the field to new uptake models in the future, including the transporters (OCT, PMAT) that have been implicated with antidepressant mechanisms and behavior responses [61, 63]. Likewise, the advent of new, flexible and implantable biosensors will allow for the chronic measurement of serotonin and its relationship to antidepressants and behaviors that will expand our fundamental knowledge of long-term changes in the near future [59].

### Histamine, inflammation, and immune cell signaling

Histamine (HA) is another monoamine neurotransmitter, similar to serotonin, that plays important roles in sleep-wake cycles, as well as memory formation and learning [11, 77]. However, it also acts as a central signaling molecule that leads to a cascade of cellular immunological responses that cause smooth muscles to contract and blood vessels to become permeable. Histamine increases inflammation and swelling with the activation of radical oxygen species (ROS), which are known to act as a signaling molecules and damage cells. Four sub-classes of histamine receptors (HR_1-4_) have been identified in mammals, all GPCRs, but they differ in their tissue expression and actions [77]. For instance, HR_1_ and HR_2_ receptors are mainly expressed in airways with vascular smooth muscle cells to activate or inhibit the release of histamine from immune cells. HR_4_ is found in bone marrow and white blood cells. HR_3_ receptors act as pre-synaptic receptors in the central nervous system and control the release of histamine and other neurotransmitters, such as serotonin, dopamine, norepinephrine, acetylcholine, and GABA [77]. HR_3_ receptors also connect neurons with immune cells, like mast cells. In addition to mammals, *Drosophila melanogaster* also produces histamine and possesses homology to these histamine receptors [78]. Histamine activates inflammation and immunological signaling and may regulate other neurotransmitters, like serotonin, so recent studies have concentrated on measuring both serotonin and histamine to determine their roles in depression.

**Fig. 6:**
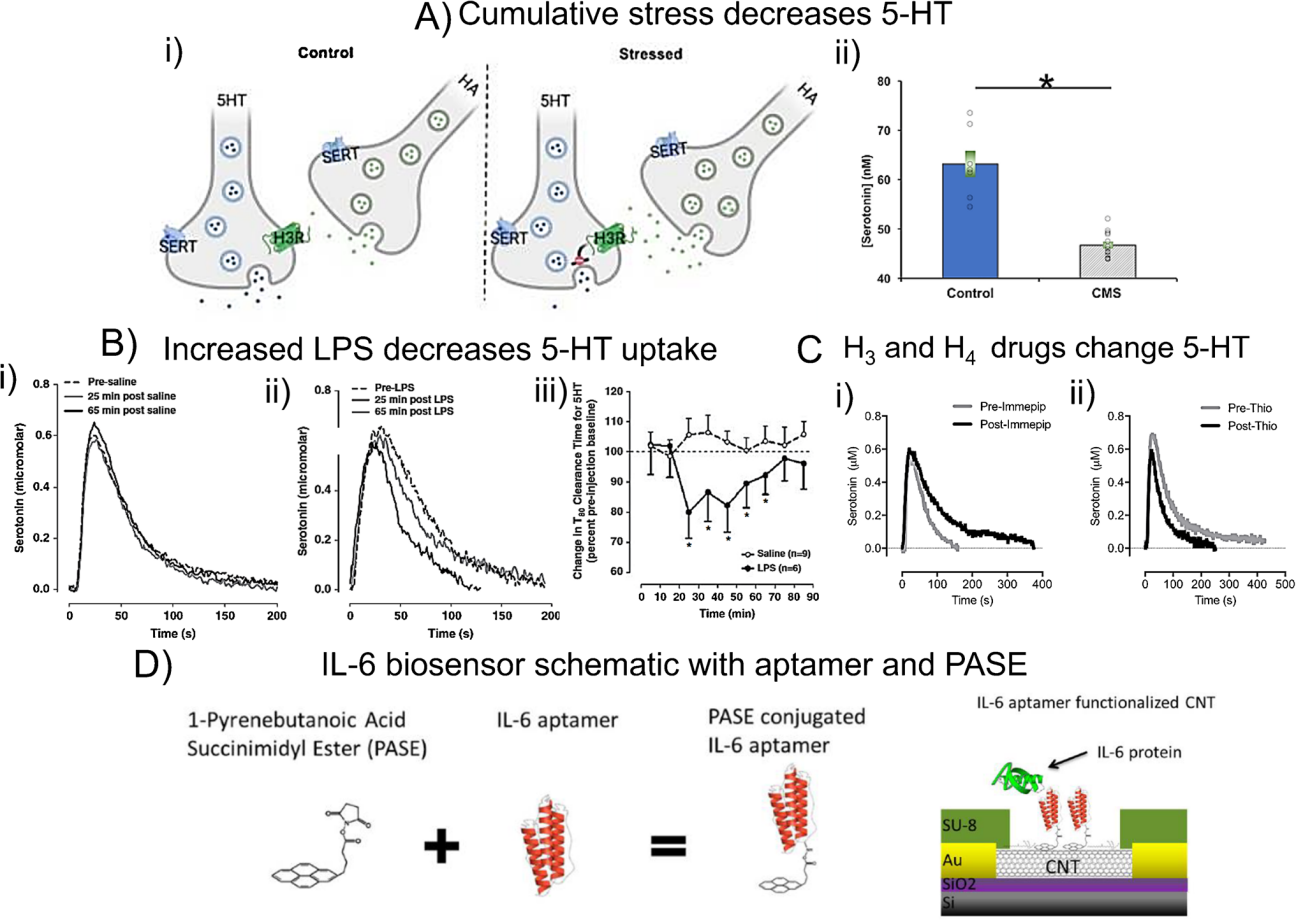
Real-time electrochemical techniques show histamine (HA) changes 5-HT. **A** With stress, increased histamine (i) causes 5-HT to decrease (ii) (permission from Hersey et al. 2022, *Journal of Neuroinflammation*). **B** Increased LPS signals for IL-6 and changes serotonin compared to the control (i). Increased LPS decreases serotonin concentration and uptake (ii) over time (iii), but returns to baseline (permission from Zhu et al. 2010, *Neuropsychopharmacology*). **C** The Daws group used chronoamperometry to measure 5-HT changes when immepip (H_3_/H_4_ receptor agonist) and thioperamide (H_3_/H_4_ receptor inverse agonist) were exogenously applied for 30 min to the mice hippocampus. H_3_/H_4_ receptor agonism slows serotonin clearance, while H_3_/H_4_ receptor inverse agonism both decreases 5-HT concentration and quickens uptake (permission from Annamalai et al. 2020, *ACS Chemical Neuroscience*). **D** Example of an IL-6 cytokine aptamer-based biosensor that could measure IL-6 changes with histamine and serotonin during depression (permission from Dutta et al. 2021, *Biosensors*)

With FSCV, the Hashemi lab developed electrochemical methods to investigate inflammation with depression. They co-detected histamine and serotonin with FSCV using the Jackson waveform in vivo in mammals with Nafion-coated electrodes (Fig. 6A) [40]. They found serotonin and histamine concentrations change with long-term stress and treatment with escitalopram [79], and that stress to mice increased histamine concentrations, while serotonin decreased. Escitalopram alone did not bring serotonin back to baseline levels in stressed mice, but concentrations did increase with combined escitalopram and α-fluoromethylhistidine (FMH), a histidine decarboxylase inhibitor. These results suggest that histamine clearance influences serotonin release and clearance during stress. The Hashemi group also collaborated with the Orian lab to test a novel selenofluoxetine derivative [80]. The selenofluoxetine derivative binds to SERT similarly as fluoxetine to inhibit serotonin reuptake with FSCV and models show that the selenium group adsorbs oxygen radicals that cause inflammation, which are mediated by histamine. Altogether, these analytical tools help us understand real-time serotonin and histamine signaling during stress and with antidepressants and contribute to our basic knowledge of the mechanisms of new antidepressant pharmaceuticals that decrease inflammatory responses.

The Daws group also used chronoamperometry to measure serotonin changes with different pro-inflammatory cytokines and drugs that impact histamine to understand their roles with depression. For instance, they studied the cytokine inducer lipopolysaccharide (LPS) and its downstream effects on SERT [81], as well as tail suspension (TST) and forced swim tests (FST) to understand changes with LPS to depression behaviors. LPS activated SERT by increasing serotonin reuptake (faster reuptake) to subtly decrease serotonin concentrations in the mouse hippocampus (Fig. 6B). With the behavior tests, LPS injections increased immobility time, which increased depression behaviors. They also investigated the effect of increased LPS on serotonin concentrations with interleukin-1 receptor (IL-1R) deficient mice and their responses by applying SB203580 (p38 MAPK inhibitor). They found SERT activation requires IL-1R and the MAPK pathway with increased LPS. Together, these data suggest that increased pro-inflammatory LPS increases SERT activity to decrease serotonin and leads to depression behaviors that are mediated by several downstream signaling cascades, including IL-1 receptors and the MAPK pathway. Altogether, the measurements of serotonin here revealed that histamine and cytokines are critical to the molecular mechanisms of depression and are possible targets for pharmacological therapies.

Likewise, the Daws group also collaborated with several groups to compare SERT reuptake regulation by histamine receptors [82]. They used in vivo chronoamperometry in several regions of the rat brain to compare SERT activity with an H_3_/H_4_ receptor agonist (immepip) and an inverse agonist (thioperamide) (Fig. 6C). In the central nervous system, H_3_ receptors act as heteroreceptors to serotonin and regulate its release, but their effect on reuptake was unknown. H_3_/H_4_ receptor agonism slowed serotonin reuptake by SERT in the hippocampus, while H_3_/H_4_ receptor inverse agonism decreased serotonin concentration and caused faster serotonin reuptake by SERT [82]. The H_3_/H_4_ agonist immepip affected SERT activity only in the hippocampus and cortex, which were regulated by the CaMKII/calcineurin pathways. Altogether, the Daws and Hashemi data are similar and showed increased histamine due to inflammation, which decreases serotonin concentrations. Ultimately, bioanalytical techniques that directly measure neurochemicals show histamine is an important neuromodulator in depression, and has profound impacts on inflammation and immune cell signaling that directly change serotonin regulation through SERT activity.

Along with this work, electrochemical biosensors have also recently become popular to directly measure cytokines, which are important in inflammation [14, 15, 83]. There are several classes of cytokines, including growth factors, interferons, lymphokines, monokines, tumor necrosis factors (TNFs), and chemokines that are described in Table 1 [83], and each plays different roles in regulating cellular responses for inflammation and/or infection. In addition to depression, consistent upregulation of pro-inflammatory cytokines is associated with other serious illnesses, such as Alzheimer’s disease, cardiovascular disease, Rheumatoid arthritis, cancer, sepsis, and COVID-19 [15, 84]. Currently, many labs are designing novel biosensors to measure how cytokines change in different biofluids (saliva, serum, urine, sweat, etc.), which will aid in future experiments to explore these as biomarkers of diseases and their treatments [84]. For example, the Panchapakesan lab created and characterized a label-free IL-6 aptamer-based FET biosensor using a carbon nanotube micro-array, which is shown in Fig. 6D [85]. The biosensor consisted of a SWCNT/SiO_2_/Si gate coated in RNA aptamers, and IL-6 interacted with the aptamer to create a conductance that allowed IL-6 to be quantified in real-time from 1 pg/mL to 10 ng/mL. Another study, from the Zhu lab, also developed a heated carbon paste electrode with an antibody immunoassay to detect IL-6 [15, 86]. Along with IL-6, several groups have also developed TNF-ɑ biosensors [15]. In the future, these micro-biosensors for cytokines could be combined with electrochemical techniques for histamine or serotonin detection to enhance research to fundamentally characterize how cytokines and ROS influence depression and other illnesses.

**Table 1:** Cytokine classes with examples and their basic cellular activities and cell types

Cytokine class	Cytokine examples	Predominant cellular activity
Growth factors	G-CSF, GM-CSF, M-CSF, and VEGF	Stimulate cell proliferation and differentiation
Interferons	ɑ, β, γ	Antiviral activity
Lymphokines	Interleukin-2, -3, -4, -5, -6, -9, and -10	Lymphocyte signaling molecules
Monokines	Interleukin-1ɑ, -1β, -12, and -15	Mononuclear phagocyte signaling molecules
Chemokines	Interleukin-8, MCP-1, MIP-1ɑ, MIP-1β, RANTES	Cell migration and activation, chemotactic
TNFs	TNF-ɑ	Cell proliferation, control, and death

Overall, these studies indicate that histamine plays an important role in regulating serotonin concentrations during depression and with chronic stress. Specifically, when histamine concentrations increase [79], serotonin concentrations tend to decrease from overactive SERT [79, 81], which is mediated by immune cell and receptor signaling [81]. Furthermore, inflammation and the creation of ROS species increases oxidative damage to cells [80], which is linked to increased depression and other serious illness [15, 83, 84]. These works show that new pharmaceuticals could bind ROS species or target SERT, as well as histamine and IL-receptors to combat chronic inflammation and improve depression symptoms. Thus, new methods to monitor histamine, serotonin, ROS species, and cytokines simultaneously would give even better insight into inflammation and how it causes depression.

### Ketamine, glutamate, and serotonin

Recent studies have also indicated that glutamate modulation plays a role in treating depression, especially with novel microdosing ketamine treatments. Trullas and Skolnick were the first to suggest that ketamine could be used to treat depression [16], since long-term stress can cause depression symptoms that disrupt potentiation in the hippocampus and is regulated by glutamate with NMDA receptor activation [16, 20]. However, ketamine also activates glutamatergic AMPA receptors, which leads to an increase in TrkB receptor stimulation and facilitates mTORC signaling with the release of brain-derived neurotrophic factor (BDNF) [19, 20]. BDNF increases cytoskeletal reconfiguration, as well as dendritic spine turnover [20]. Together, these changes increase neuroplasticity in the prefrontal cortex (PFC). In addition to glutamate, ketamine may also have effects on serotonin receptors and SERT, but the evidence is less clear [33, 87]. However, since ketamine is a “dirty” drug that interacts with many molecular targets [20], it is difficult to pinpoint specific mechanisms with these neurotransmitters and signaling molecules.

Glutamate is the most abundant and major excitatory neurotransmitter in mammals [88, 89], and is regulated by a variety of transporters and receptors [88]. The transporters are subdivided into two major classes: excitatory amino acid transporters (EAAT) and vesicular glutamate transporters (VGLUT) [88]. In depression research, the EAAT glutamate transporter-1 (aka EAAT-2 transporter) has been heavily implicated in changing glutamate concentrations that impact behavior [90, 91]. In addition to transporters, glutamate receptors are classified as ionotropic and metabotropic [88]. The ionotropic receptors include NMDA, AMPA, and kainite receptors, while the metabotropic receptors include Group 1, Group 2, and Group 3 receptors. NMDA and AMPA receptors are major interests with micro-dosing ketamine [20]. Although glutamate is an excitatory molecule in mammals [92], it shows excitatory and inhibitory effects in *Drosophila* in their neuromuscular junction [93] and olfactory system, which complicates it as a model [94]. Over the past decade, several works have used biosensors to understand glutamate changes with depression and chronic stress, especially in mice models [90, 91, 95, 96].

**Fig. 7:**
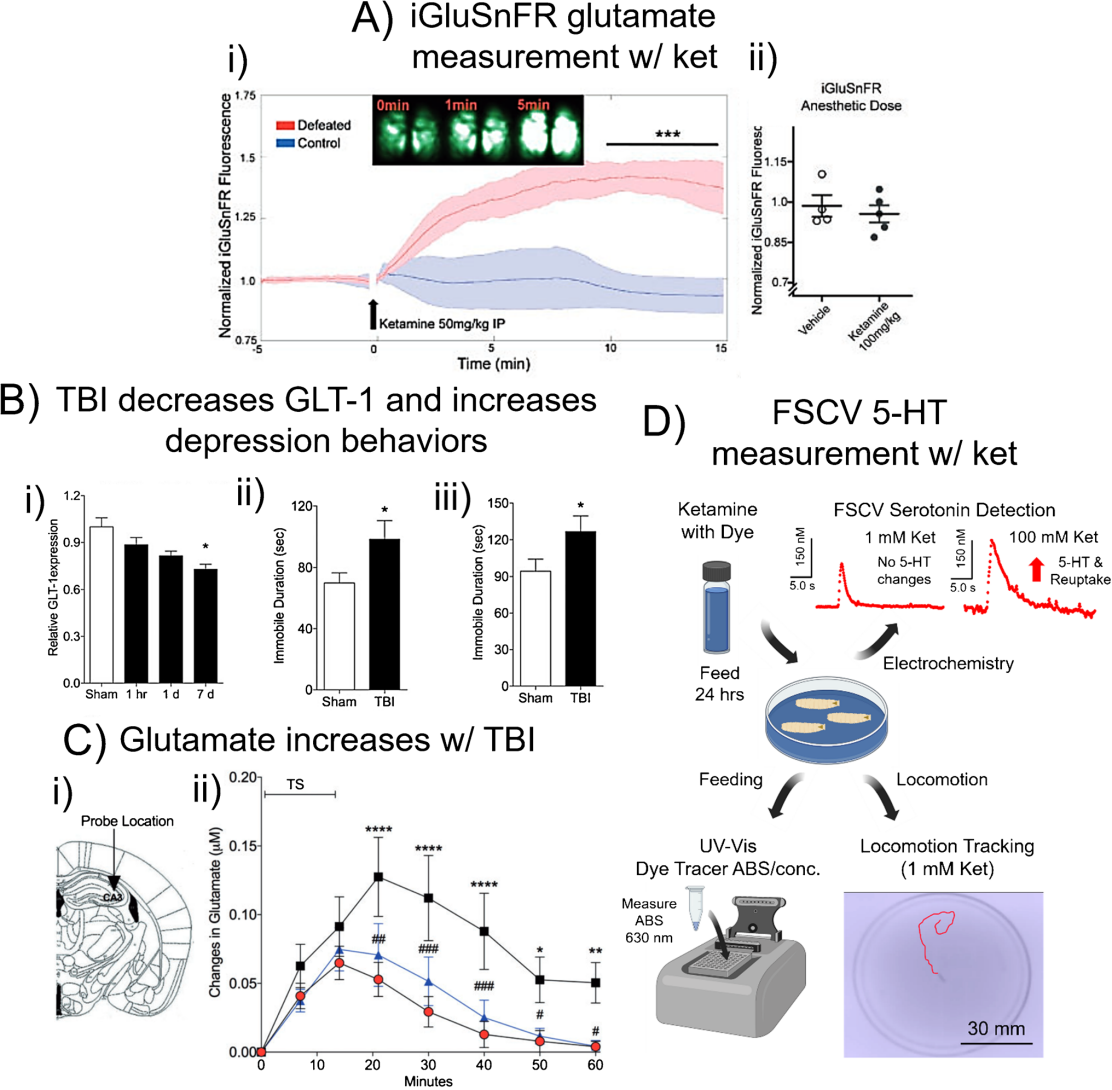
Measuring glutamate and serotonin changes with ketamine and depression. **A** The genetic sensor, iGluSnFR, was used to measure glutamate changes with (i) sub-anesthetic and (ii) anesthetic doses of ketamine. Glutamate increases with lower doses in a stressed mouse model, but not at anesthetic doses (Permission from McGirr et al. 2017, *Brain*). **B** Traumatic brain injury (TBI) decreases expression of the (i) glutamate transporter (GLT-1), and increases depression behaviors in mice using the (ii) tail suspension test and (iii) forced swim test (permission from Piao et al. 2019, *Journal of Cerebral Blood Flow & Metabolism*). **C** (i) Glutamate biosensor developed by the Wilson and Petillo labs was inserted into the mouse brain for in vivo glutamate measurements. (ii) Stress mouse model (black) increased glutamate and did not return to baseline. Stress mice given fasudil (ROCK pathway inhibitor, blue) decreased glutamate. Sham model (red). **D** Real-time 5-HT FSCV measurement in *Drosophila* larvae. 5-HT does not change with micro-doses of ketamine, but slows uptake and increases concentrations with a high, anesthetic dose. Feeding and locomotion behaviors also change depending on dose (Dunham et al. 2023, BioRxiv. preprint)

New glutamate sensors, like iGluSnFR, have become useful to explore real-time changes in specific brain regions with ketamine and depression [90, 91, 96, 97]. For example, the McGirr and Murphy labs used iGluSnFR to measure glutamate changes with a chronic stress mouse model (chronic social defeat), and found that glutamate increased in the stressed mice [96]. Additionally, glutamate changes with sub-anesthetic versus anesthetic doses of ketamine and they saw that glutamate increased in the stressed mice at a low dose (Fig. 7A), but did not change with the higher dose. Another study used iGluSnFR to measure glutamate changes in mPFC astrocytes with genetic mutations to the O-GlcNac transferase (OGT) enzyme, a key protein in O-GlcNacylation, to understand glucose metabolism dysfunction and depression [91]. They found that OGT modulated glutamate transmission by interacting with GLT-1, and that OGT astrocyte specific knockout mice showed decreased stress and lower glutamate concentrations. The Wainwright lab also investigated glutamate and depression behavior changes following traumatic brain injury (TBI). Long-term TBI decreases expression of GLT-1, as well as increases depression behaviors in mice using the TST and FST, which are shown in Fig. 7B. They also used a glutamate biosensor developed by the Wilson and Petillo labs [98] to measure glutamate in vivo with changes to specific signaling pathways, PAR-1 and ROCK (Fig. 7C). Glutamate concentrations increased with the chronic stress mice, but decreased with the ROCK inhibitor, fasudil, and was similar to the control sham mice. Together these results suggest multiple new pharmacology targets, including the O-GlcNac and the ROCK pathways, which could be explored for depression treatments in the future.

Although it is not possible to measure glutamate directly with electrochemistry, serotonin changes can be electrochemically measured after ketamine. Recently, the Daws group used chronoamperometry to measure serotonin changes in SERT double knockout (-/-) mice and found that high doses of ketamine (32/mg/kg) slowed serotonin reuptake [33], which led them to conclude that SERT inhibition was required for ketamine’s antidepressant effects. However, they only measured serotonin changes with a single, high dose and did not investigate lower doses similar to a micro-dose treatment. More recently, the Venton lab used FSCV to investigate serotonin and behavior changes with various doses of ketamine and SSRIs in *Drosophila*, which is illustrated in Fig. 7D [99]. Here, they found that serotonin dynamics did not change at a low, 1 mM feeding dose, but concentration increased and uptake was inhibited at a higher, 100 mM dose, which is similar to the data collected from the Daws lab [33]. The 1 mM ketamine dose also increased feeding and locomotion, but the 100 mM dose (which is anesthetic) significantly decreased these behaviors [99]. It is important to note that these results in flies are similar to a previous study where PET imaging was used in humans to understand SERT inhibition with micro-dosing ketamine [87]. They also did not see SERT inhibition at lower doses, but suggested higher doses may block reuptake. Likewise, the Hashemi lab investigated changes to serotonin using FSCV and FSCAV in vivo in mice [100]. Here, they found that basal levels of serotonin slightly increase over several hours with ketamine, but ambient release does not change. In addition, they found that histamine negatively modulates serotonin release with ketamine.

Altogether, these electrochemical studies suggest serotonin dynamics may not change with ketamine micro-dose treatments, and demonstrate that ketamine does not work primarily on serotonin for its antidepressant effects. Still, these results show the value of directly measuring neurotransmitters to determine targeted and non-specific effects of ketamine. Future work should also concentrate on glutamate measurements after other antidepressant treatments to determine if this is their main mechanism of action.

Ultimately, these studies indicate that glutamate and serotonin play differing roles with depression and ketamine treatment. Interestingly, several groups showed that chronic stress increased extracellular glutamate and decreased GLT-1 transporters [90, 96]. However, compared to previous data from the Hashemi lab [79], serotonin concentrations tend to decrease with chronic stress and increase pro-inflammatory immune cells with histamine. In addition to these works, new research has also explored how ketamine changes cytokines [101–103], and that cytokine regulation may play a role in TRD [101, 102]. Thus, future research could measure glutamate and cytokine changes with biosensors to understand how they change with stress, behavior, and ketamine or other antidepressants. Additionally, serotonin did not change with micro-doses of ketamine, but new research with electrochemistry and glutamate or cytokine biosensors could inspire new work to try to understand their signaling with other common antidepressants, like SSRIs, or the creation of new pharmaceuticals that target all of these neurochemicals.

## Future multi-neurotransmitter detection tools and applications for complex depression mechanisms and treatments

Although these individual electrochemical techniques help to identify neurotransmitter changes with these different depression hypotheses, future work would benefit from combining them to understand how multiple neurotransmitters change with depression. For example, although chronoamperometry and FSCV have millisecond temporal resolution [22], they lack specificity and spatial resolution since their microelectrodes are still too large to sample from the synaptic cleft [22]. Therefore, fluorescent genetic sensors, such as SnFRs or GRAB sensors [27, 29], would be useful to image neurotransmitters in specific locations in the body, while amperometric or voltammetric techniques measure them with high sensitivity. Additionally, these biosensors will also help with the simultaneous detection of non-electroactive neurotransmitters and messengers, like glutamate and cytokines [15, 83, 90, 91, 96], and the regular canon of electroactive neurotransmitters implicated in depression (serotonin, histamine, and dopamine). Further, these works show that FSCV can be made more specific with new waveform designs to specifically measure one neurotransmitter over another [37], which could be combined with other electrochemical techniques to accomplish specific, multi-analyte detection with several electrodes or biosensors [104].

In addition, these tools could be combined and optimized with new in vivo setups in mice and flies in order to understand neurochemical changes with behaviors, such as feeding, locomotion, or sleep with antidepressant use [40, 68, 105]. Although in vivo electrochemistry changes to serotonin in humans has been minimal [51], momentum has increased in the last few years to improve long-term electrode implantation to study neurotransmitter changes in humans, as well as create wearable biosensors that measure biomarkers for depression (i.e., serotonin, histamine, and cytokines) in fluids, like sweat [15]. Likewise, chemical diagnostics for depression can improve by using pluripotent stem cells to understand genetic effects with antidepressants to achieve more personalized treatments [106]. Indeed, future research will progress soon to create better, easier to use devices for diagnosing depression, as well as personalized treatments for it.

Ultimately, this review shows that analytical techniques are helping to define the mechanisms of the many chemical hypotheses for the etiology of depression and that multiplexed measurements of serotonin, histamine, cytokines, and glutamate would provide a better picture of the complicated disease that is depression [11, 19]. All things considered, combined techniques would allow for faster drug and behavior screenings [75] and enhance our understanding of current treatments to make them better and improve their efficacies.

## Conclusions

Depression is a common and debilitating illness with unknown etiology and unclear neurochemical changes. Over the past few decades, several hypotheses have been developed that focus on the neurotransmitter changes with serotonin, histamine, and glutamate, as well as immune mediators, like cytokines. In addition, the most common treatments for depression, including SSRI antidepressants, are extremely variable, and new micro-dosing ketamine therapies are not well understood. However, improved electrochemical techniques have made it possible to investigate antidepressant mechanisms, as well as genetic and downstream cellular effects. Electrochemical techniques, like chronoamperometry and FSCV, have deciphered real-time serotonin and histamine changes with SSRIs and other serotonin and histamine receptor drugs. Additionally, biosensors have become very popular over the past few years to measure real-time changes in glutamate and cytokines, which are not electroactive. The studies described in this review show that depression is a multi-faceted illness, with many neurochemical targets that also interact with each other. Analytical techniques will define new pathways and druggable targets that show the potential for new pharmaceutical development. Ultimately, these studies highlight individual mechanisms for depression and antidepressants, but also motivate future research that will need to combine and multiplex them in order to understand the molecular causes of depression and create better treatments for it.
